# The Life of the Trichina

**Published:** 1868-12

**Authors:** Rudolph Virchow, Rufus King Browne

**Affiliations:** Prof. University of Berlin


### Original Communications.

### THE LIFE OF THE TRICHINA.

BY RUDOLPH VIRCHOW, M.D.PH.D., PROF. UNIVERSITY OF BERLIN.

Translated by Rufus King Browne, M. D.

[Continued from page 461.]

### 4. WHAT PREVENTIVE MEASURES ARE NECESSARY AGAINST THE SPREAD OF THE TRICHINA.

In the first part, I named those animals in which Trichina had hitherto been found, from which we see that carnivorous and omnivorous animals are mentioned. The mole was named, which most farmers think to be only a plant and root eating animal; but it is of importance as a destroyer of grubs, rain-worms, snails, mice, and young rats, as the late Gloger in his valuable writings has explained.

It is evident that in the common course of affairs Trichina can only be found in carnivora. For we have seen that the intestinal Trichina produce living young, which migrate into the flesh, and ONLY HERE ATTAIN THEIR FURTHER DEVELOP-

1. Gloger. The useful friend of the farmer and forester among the animals. Instruction to Farmers and Schoolmasters. Berlin, 1859, p. 9. Injunctions to protect useful animals which prevent mice and other vermin. Berlin, 1862, p. 7.

MENT. P. 29, and that they can not leave the meat in any other way than after it has been eaten. This regular progression from the intestine into the muscle, and from the muscle into the intestine, is only possible in meat eaters. Still there are two exceptions.

In the first place, the plant eating animals, are not so perfectly select in the choice of their nutrition, that they might not occasionally swallow meat. If small pieces of meat are placed in the mouth of a rabbit, calf or pigeon, it swallows them, and upon this depends the possibility of feeding them with Trichina meat, of which we have spoken. It might happen, therefore, by accident, that without artificial feeding, a plant animal might eat Trichina meat. Still, for the consolation of the reader, it may be said that such cases have not been observed.

There is another way imaginable than the eating of meat, by which an infection may occur. Leuckart[1] has pointed out this possibility. If, for instance, in an animal, the intestines of which contain pregnant Trichina, and the contents when expelled, of the intestine of which are eaten by a second, the latter may become infected. It is known that pigs will eat human excrement, and this infection may occur without the eating of meat. Although this kind of infection has not yet been proved, it is very likely that it may occur in pigs. And it can not be denied there is a possibility of other animals also being affected in this way.

It is not certain that the Trichina found in moles, flesh-eating birds, or even cats, are identical with those found in human beings. If we leave out of consideration that in large cities cat's meat is sometimes eaten, the Trichina found in the former animals, are of no interest in our investigation, except that pigs might be infested from them. For this we have as yet no certain data, and it might be a subject of further investigation only for the purpose of ascertaining where the moles get their Trichina from.

---

1. Leuckart. See p. 50.

I CONSIDER IT AS ESTABLISHED THAT IT IS ONLY THE UNCLEAN OR CARNIVOROUS ANIMALS, THAT ARE DANGEROUS TO MAN, AND THE MOST DANGEROUS AMONG THESE IS THE PIG. It is not yet known whether the wild boar is liable to infection. THE PLANT EATING ANIMAL CAN BE CONSIDERED CLEAN and not dangerous. Recently it has been rumored that some of Trichina infested pigs had been raised in the Abdeckerein, and fed on meat of killed or died animals. This is not only untrue, as a fact, as I have ascertained on inquiry in Hettstedt and other places, but is also theoretically erroneous. Because the meat which is found in "Abdeckerein" is of horses, cattle, and sheep.

THE MAIN CARE OF MUNICIPALITIES AND INDIVIDUALS, SHOULD BE DIRECTED TO THE PIGS. The following points of view are to be considered here:

1. THE INFECTION OF PIGS BY TRICHINA HAS TO BE GUARDED AGAINST.

I mention here, that an originating of Trichina in pigs does not occur. Trichina are procreated like men, from father and mother. They multiply by legitimate progeny, and their being found in an animal pre-supposes INFESTATION from without, *i. e.* by means of FOOD.

In all cases of prevention it is necessary to examine the food of the pigs, and to allow them no opportunity to devour refuse animal matters, as such matters are mostly Trichina infested meat, and excrements of individuals infested by Trichina, particularly human excrements. Feeding of the pigs in styes scrupulously clean (which is really the interest of the owner,) would give the greatest security, although of course infections by chance might not be precluded.

Whether fattening on acorns in forests precludes infestation is not certain; experiences are wanting, and the fact that wild animals have had Trichina leaves presumed security questionable.

Careful farmers and raisers of animals will see the importance of the above suggestions. Most of the epidemics of

Trichina disease have occurred in districts of Saxony, where it is the rule to feed pigs in styes. We can only recommend the most scrupulous cleanliness.

2. All meat ought to be carefully examined. We said before, there is no certain sign of Trichina disease in pigs. Nothing will suffice but a careful investigation of the meat.

Only in a few of those in which the Trichina is encapsuled and encrusted in lime, is the investigation with the naked eye sufficient to detect their presence; most cases require microscopic examination.

For this purpose, though the best instruments are preferable, they are not strictly necessary. Microscopes of medium magnifying power are sufficient, still I call attention to the fact, that poor microscopes which pretend a large magnifying, are less reliable than good instruments of much less power. I recommend to prepare the meat as already mentioned, and examine it with a low power; if any suspicious point be observed, it should be marked, and a higher power used to resolve it into its particulars.

Instruments which by their construction for the purpose of demonstration give a diffused light, do not give so sharp a view of the contour of a soft object, as is desirable to the unpracticed observer.

The question arises, *who* shall investigate the meat?

I reply: in cities an investigation of the meat should devolve upon the city authorities, and be done by selected physicians, veterinary surgeons, or others acquainted with natural science.

The most simple way in large cities, is to have public slaughtering houses; much may be said in recommendation of these, irrespective of Trichina meat, and they have been constructed in different places in Germany. By the employ ment of these houses, a source of great uncleanliness in gutters, yards and houses could be stopped. If a city has these, nothing is easier than to have a microscope there, and allow no pork to leave it for sale, until after proper examination of the meat, testimonials had been given of its wholesomeness. The chief investigator should examine a part of several muscles of each animal, which can be done in ten minutes, and then give his testimonial of the case.

In small cities where no slaughtering houses exist, the appointed examiner should have an opportunity to investigate the meat, and I do not doubt that this is everywhere possible. Even now, the butchers at different places, Stedtein, Nordhausen, have made contracts with certain physicians, who on their part investigate the meat and attest to its purity. But this is not sufficient, for it should be undertaken not merely in the private interest of the butcher, but the public health and welfare is concerned; and for this, the State should be, under the circumstances, responsible.

Beside cities there are villages, country towns, hospitals and other institutions, ships, etc., and nothing is easier than to find a suitable person, as a teacher or physician, a clergyman or the master of a ship, to become acquainted with the means of conducting such an investigation. On larger estates, it is to be expected that the proprietor or his steward, will have sufficient interest to convince himself of the purity of the meat his workmen and families consume; and neither the labor nor price of the means of doing it, can be deemed a ground of objection in comparison with life and health.

Once more I point out that it is futile to say that the cases of disease are too infrequent to justify such expenditure of means to prevent it throughout the land, and indeed the world. What a single person will do for himself is his own affair; but the community is under obligation to prevent dangers which the single individual otherwise may in cases incur; and to assist those who may undesignedly be the means of endangering others, or where it is necessary to control them, that they may be under special requisition to exercise their trade without injury to others. If a butcher of a cow is the cause, even unwittingly, of hundreds of persons falling sick and some dying, can not complain if he is controlled in the same way as a manufacturer who deals in dangerous chemicals.

The worst is of the small country farmers, who neither possess a microscope nor are in the neighborhood of any person who is qualified to make the examination; but certainly the time will arrive when every teacher will possess a microscope. Till then, the small proprietors can only be guarded against infection by being very careful about the preparation of their food. This point we will further dilate upon.

3. THE PORK SHOULD BE COOKED VERY CAREFULLY. In some places pork is eaten raw, scraped. I do not mean merely butchers and cooks, nor merely the use of scraped meat advised by physicians, since they prescribe not raw pork, but beef; but in some places it is a common habit to eat raw pork. In Burg, a great many cases of disease and death were occasioned by people eating raw scraped meat on bread for breakfast; cases which are very striking, since sometimes in the same family single members who had eaten of the same meat cooked or fried, which others that fell sick had eaten.

We must therefore recommend *never to eat pork raw.* For even a microscopic investigation will never give an absolute guarantee, since single Trichina may have been overlooked. And if even a few enter the body without fatal results, it is still better to avoid all danger. Whoever wants to eat *raw* meat as a regimen or preference, should eat beef or mutton.

But even the cooking of it does not afford any security, unless it is done carefully. In boiling, roasting, frying or smoking, more or less of the meat may remain nearly raw, and therefore be dangerous. The greatest danger is from ham; especially, since it is cured so rapidly. In this case the ham is either not at all smoked, or that for only so short a time, that the greatest part of it remains fresh. Sometimes it is merely brushed over with creosote, wood-acid, or some other, which gives it the smoky taste, and is then offered for sale. If it contained Trichina, they remain after this preparation of the meat, entirely alive.

Formerly it was otherwise; then the pigs were killed in the fall. The hams were hung in the chimney or smoking house, and remained there to be eaten several months later. Under this treatment the Trichina are dead and harmless, but then the ham is dry and hard and is not so savory. Our forefathers did not think this an evil. They knew that a person eats less of this ham, because one is satisfied with less of it. They had the same opinions as the people of Norway, who do not smoke their meat, but let it dry for six months or a year.

Such old-fashioned ham is not to be found in trade now-a-days. Even in Westphalia, the new method is in use. The requirements of trade do not leave any on hand.

Whoever cures the ham which he will consume, may allow it to remain as long as he pleases, to preclude all danger; and therefore in the country and in small cities, where families cure the hams they consume, less danger is to be apprehended; but those who buy hams have only two possibilities for security. They must have the ham investigated by a microscope; for this it is sufficient to cut out from different places small pieces and have them examined, or they must use only well boiled ham. In the Southern part of Germany only cooked ham is consumed, which explains why until now, but few cases of Trichina disease have been known. Still they are not wholly wanting. I have several times found at Wurtzburg numerous encapsuled Trichina in human bodies, and in Tubingen and Heidelburg single cases have been repeatedly observed.

Next in danger to ham is pork sausage, specially ham sausage; liver and blood sausage should be excepted if they are carefully prepared. But sometimes fine meat is mixed with the liver and blood, and experience has shown that such sausages have caused severe disease (Dresden, Calbe, Burg). In Hettstadt it was the so-called Head-cheese in which numerous Trichina were found. In the preparation of sausage a change similar to that in the preparation of hams has lately taken place. Formerly the sausage was boiled more to make sausage soup. The long sausage ("brutwurst,") was longer fried, and the smoked sausage was longer smoked, and longer kept. But now-a-days, especially in cities where every thing is ready made for sale, everything has to be made very fast; and sausage has to be fresher or rawer, and more savory, for such is the taste of the consumer. Accordingly it is easily understood, that since the preparation of sausage has ceased to be a domestic work and come into the hands of tradesmen, the danger has increased; and this fact may explain what seems so astonishing to the public, namely, that the Trichina disease is so frequent now, if we admit that it *is* now more frequent than formerly.

But such danger was unexpected. According to an account of Dr. Rupprecht, in Hettstadt sausage was prepared as follows: the pork and its rind were boiled from 1½ to 2 hours in a kettle, was then minced and filled. The sausage was then boiled from ½ to ¾ of an hour. A portion of this sausage fried in a pan until the fat drained off, was eaten by a family of five persons. All of these persons soon fell sick, and one, a young boy died. It was ascertained that no more of the diseased pig had been eaten by them.

It is easy to understand that after this and similar experience, the population of Hettstadt were panic stricken, and the municipality as well as the administration in Merseburg, announced publicly that even boiling of the pork afforded no security.

I will return to this point presently, but meantime remark, that Dr. W. Müeller ascertained on inquiry that in making the sausage of the boiled meat and rind, *raw* minced meat was added; and the sausage was afterwards scalded, but not really boiled. These facts suffice to show the insecurity of eating sausage, especially pork, of which it is not known to the consumer how it was prepared. And the question arises whether the so-called sausage poison, as in the case of ham poison, can not be explained by the presence of Trichina.

In Swabia many cases of poisoning by sausage have been recorded, wherein chemical analysis failed to detect any poison. But I revert to the boiling and roasting. It is certain that a Trichina exposed to the boiling point 80° Reamur, invariably dies.[1] This is even the case in a temperature which will coagulate the white of egg, 50 to 60° Reamur. But it is equally certain that frequently the former temperature is not reached in boiling and roasting, or if reached, not the whole of the meat is exposed to it. This is certainly the case when large pieces are boiled or roasted; and we even see in cut slices that some is half or wholly raw. The blood and albumen have not coagulated as in the case of boiling heat. The parts are yet soft, fresh and red. The same is the case of chops. There can be no doubt that the Trichina in the inner part of the meat have not been reached by a killing temperature; and by *such* broiling, roasting or frying, the danger is not prevented.

About these relations (of heat to animal life) direct experiments have been made. Kuchenmeister[2] found that large pieces of meat, which had been put in a kettle after a boiling of ½ hour, attained a temperature of 48° Reamur. Inside it had only 44°. After boiling longer than ½ hour, the meat had a temperature of 62° to 64° outwardly. And after being cut up and put to boil they attained inside, after one hour's boiling, a temperature of from 59° to 60°. Fried sausage and chops reached a temperature of 50°. Frankfort sausage 51°. Roast pork, which was inside bloody, had 52° Reamur.

---

1. Fiedler, Arch. for Medical Knowledge. Year v., p. 57.
2. Kuchenmeister. Zeitschrift. II. p. 314.

These numbers are not fixed for all cases, and it will often happen that the temperature of the meat and sausage remains several degrees under these stated numbers.

Fiedler found that Trichina can bear a temperature of from 30° to 40° Reamur, and that even in that of 50° to 52° Reamur they do not die immediately, although they may not live long afterwards.

From this record it is evident that the common boiling or frying of sausages, as well as the preparation of chops and rare roasted pork, scarcely reaches the temperature which will kill the Trichina. I conclude with the results of trials which Kuchenmeister, in conjunction with Huebner and Liesering,[1] made.

1. The Trichina are killed by longer immersion in brine of the meat, and by 24 hours hot smoking of sausages.

2. They are not killed by three days' cold smoking, and it seems the boiling of meat for making sausages does not certainly kill them.

3. A long keeping of cold-smoked sausages seems to destroy the life of the Trichina.

Let every one now reflect how far the foregoing facts shall influence him. My task was not so much to cause fear and to agitate the community more than it already is, but rather to show the means of shielding themselves from unquestionable danger.

These are conditions which the police alone can not prevent, but each individual must protect himself. But to do this, he must understand the details of these conditions, and it seems to me, notwithstanding the many fold and widespread communications concerning the subject, that only a continuous and connected exposer can clear up all doubts. If I have succeeded in this, my purpose is attained, for it is the noble calling of science that it heals the wound it inflicts.

---

1. Helmin theologische Versuche. p. 8.

### APPENDIX.

Although, in the foregoing, the main points of the subject have been sufficiently elucidated, various enquiries and the comprising resolves of the Land Bureau, have induced me to enlarge further upon it. Besides, there are some new facts to be stated.

There is a wide-spread fear that other meat, especially that of plant feeding animals, may contain Trichina. The administration at Merseburg have publicly announced that beef was not exempt from Trichina. According to our knowledge this fear is wholly devoid of foundation. I am acquainted with but a single fact which might induce this suspicion. In the epidemic of Calbe, some sick persons, of which, however, it was not ascertained that they were infested with Trichina, stated that they had only eaten beef. But they stated this after having been sick some time, and how unreliable statements under such circumstances are need not be told. As yet, Trichina has never been observed in beef. If it has been ascertained that the Trichina disease was contracted from consuming beef obtained from the butcher who sold Trichina containing pork, it must be ascertained whether the former had not become infested by lying in contact with the latter. This might happen in a store where both kinds of meat were sold, and the fact should be regarded as a strong argument for the institution of public slaughtering-houses and investigation of meat.

I know there are some difficulties in the accomplishment of the latter, especially that of the expense. Its great importance, however, should outweigh such considerations, when the case of the public welfare is in question. If general slaughtering-houses for all the animals consumed as food are too expensive, we should at least have one for pigs. A great source of impurity of air and soil would be removed from cities, especially the drenching of the soil with putrid matter, which increases in large cities at a fearful rate.

No other animal is slaughtered in such numbers as pigs. In Berlin the yearly consumption is 100,000. It is worth while to interfere in this matter. The other slaughtered animals, although they may have dangerous diseases, do not endanger the life of man. Neither in mutton nor beef have Trichina been found, nor are those animals liable to them.

Careless observers will, of course, find Trichina every where. There is a considerable number of small, round worms which, without being Trichina, have, in their size, and shape, and their appearance, a likeness to Trichina. Formerly one called every round worm found in meat, and which was small and undeveloped, and, perhaps, rolled up, a Trichina.

Even in eels,[1] as well as in the frogs,[2] the primitive bundles of muscles contain round worms, which are very similar to the Trichina, although they can not be regarded as the same animal. Langenbeck, in Hanover,[3] has lately maintained that Trichina are found in various lower animals, especially in great numbers in the rain worm.

And that pigs which are much in the open air, like the Hungarian, are infected by the rain worms they eat. It is not, however, found that the unconfined pigs, especially the Hungarian, are, in comparison to others, more frequently infested. Now, it is not ascertained that the rain worm has any Trichina. Certainly it has the very common microscopic round worms. That, to the older observers[4] known as Acaris Minutissima Microscopia, is as little a Trichina as that known to the recent observers[5] as *Dicetis*. At a very

1. Bowman Cyclopedia of Anatomy. p. 512.

2. W. Kuhne. My Archive. Vol. XXV. p. 222. Aberth. Feitschrift for Scientific Zoology. Vol. X. p. 530. Table XXXV.

3. Max. Langenbeck. Allgemaine Med. Zeitung of Vienna. 1864. . I. p. 6.

4 Johann. Aug. Ephr. Goetz. Attempts at the Natural History of intestinal worms in animal bodies. Blankenburg. 1782. p. 110. Tab. IX. Fig. X.

5. Dissing, Revision of the Nematoden, A. A. O. p. 627.

recent day I have, with Dr. Gentecker, made comparisons and found the most essential differences.

Even less consideration can certain free living round worms claim. I hear from several persons that the frequency of the Trichina disease in the counties of Saxony comes from the feeding of beets. In fact, Schact[1] stated that on the roots of the sugar beet Trichina were found, but that this designation is more than a common mode of expression, I must doubt.

These worms may have an injurious influence, as, after feeding oxen with bad beets, epidemics have appeared among these animals and caused the death of numbers of them.

It seems almost certain that the infestation of pigs by Trichina arises from their consuming fœcal matters, and that in a country in which the disease is more frequent, at all times particular individuals or pigs were infested, from whom the disease spreads. In regard to this I have reinvestigated the facts and found many points in support of the conviction.

In some localities there seem to exist real endemics. In the district of Marburg we have observations from the year 1845. In Hamburg to 1851; and even these places are now much exposed. In March, 1862, there was a great epidemic in Plauen. A second in the neighborhood of Falkenstein, May, 1863, and again in Plauen[2] in September of the same year. In Magdeburg the cases of this disease stretch over a period of four years.[3] The most characteristic is the appearance of the disease on the Island of Rugen. The first known epidemic was in the year 1861, when Trichina pork was sent from an estate on the peninsula Jasmond, to three other estates northwest of Gentz, viz: to Pluggentin, Muhlitz and Bergelase, and in all these places cases of dis-

1. Canstadt Yearly Report for 1862. Ed. by Scharer, Virchow and Eisenmann. Wurtzburg. 1863. Vol. V. p. 12.

2. Koenigsdoffer Deutsche Klinick. 1863. No. XLVII.

3. Zendler Deutsche Klinick. 1863. No. II.

ease appeared. In January, 1863, new cases appeared at Spyckker, on Jasmund, and recently Dr. Holthoff informs of a small epidemic at Gleselitz, south of Gentz.

The transportation of the disease is undoubted, and the supposition is most probable that there are certain (healthy) ways from which the new disease and transportation of disease proceed. If a man eats Trichina meat and the excrementitious matters are devoured by a pig, after a certain length of time, the danger of becoming sick will again be in operation. Generally six months or a year will pass before these pigs will be butchered.

This possibility should be taken in account in any investigation of meat for communities or individuals. Perhaps it will be possible in this way to remove an obscurity which yet hangs over the Trichina disease. In investigating the ham and the sausage which are received from Userlitz, a very small number of Trichina were found, and it required a very close scrutiny to find these.

I am led by this to mention that the investigation of sausage is dubious, if one is not sure that the sausage is made of clean meat. How is it possible to say that a sausage is clean if we do not know whether it is made of meat from one and the same animal, and if in the upper part of the sausage the meat is not the same as in the under. In ham it is different. Here one investigation is sufficient, and if the butchers would only sell ham which had been officially declared sound, there would be sufficient security. For such an investigation we should take slices of meat an inch long and one-half inch in breadth, for so only can we exclude the chance of cutting between the Trichina. In the ham from Userlitz there was, in a slice of these dimensions, only one or two Trichina. The cut should be made in the direction of the fibres, and not sideways or crosswise, so that the observer is able to look over a large extent of the same fibre. Finally one should lay the meat very straight on the side, because otherwise single fibres might be contorted and appear to the inexpert as Trichina or round worms.

In conclusion, I mention that pure bacon is, according to all experience, not dangerous and may be eaten without harm. The same of all inner parts not containing muscle, as brain, liver, kidney, etc.

